# Longitudinal associations and mechanisms linking frailty, depression, and self-perceptions of aging among community-dwelling older adults: a psychosomatic interaction perspective

**DOI:** 10.3389/fpubh.2026.1880962

**Published:** 2026-07-13

**Authors:** Yongwei Liu, Yuyang Luo, Junjun Sun, Huimin Zhang, Yanyan Luo, Guodong Wang, Guiying Yao

**Affiliations:** 1 School of Nursing, Henan Medical University, Xinxiang, Henan, China; 2 Xinxiang Key Laboratory for Chronic Disease Basic Research and Intelligent Care, Xinxiang, Henan, China

**Keywords:** community-dwelling older adults, depression, frailty, psychosomatic interaction, self-perceptions of aging

## Abstract

**Objective:**

To investigate the longitudinal dynamic relationships among frailty, depression, and self-perceptions of aging in community-dwelling older adults, to analyze the mediating role of depression between frailty and aging cognition, and to provide a theoretical basis for physical and mental health interventions for this population.

**Methods:**

A longitudinal follow-up research design was adopted, involving three waves of surveys (T1, T2, T3) conducted with 286 community-dwelling older adults. Assessments were performed using the Frailty Phenotype, a depression scale, and a brief version of the Self-Perceptions of Aging Scale. SPSS 26.0 was used for descriptive statistics and correlation analysis, while Mplus 8.3 was employed to construct cross-lagged panel models. Covariates such as age, gender, education level, the number of chronic diseases, and cognitive function were controlled.

**Results:**

The cross-lagged panel model demonstrated good fit indices. With the exception of baseline (T1) self-perceptions of aging not significantly predicting the midterm (T2) level, all other core variables exhibited significant temporal stability. Controlling for autoregressive effects and covariates, significant bidirectional predictive relationships emerged: T1 frailty significantly predicted T2 depression, and T2 depression significantly predicted T3 frailty; T1 depression significantly predicted T2 self-perceptions of aging, and T2 self-perceptions of aging significantly predicted T3 depression. Furthermore, the direct predictive path from frailty to self-perceptions of aging was not significant, suggesting that depression may constitute an indirect pathway linking frailty and self-perceptions of aging.

**Conclusion:**

This study supports a dynamic psychosomatic interaction pattern among frailty, depression, and self-perceptions of aging in community-dwelling older adults. Depression serves as a core hub connecting physiological frailty and subjective aging cognition. The findings support the Psychosomatic Interaction Theory (PIT) and Stereotype Embodiment Theory (SET). These results suggest that community nursing practice should emphasize early screening and intervention for depressive symptoms. Targeting this potentially important depressive pathway may help interrupt the adverse cycle of “physiological frailty – psychological depression - negative aging cognition” among older adults can be disrupted.

## Introduction

With the rapid acceleration of global population aging, the health issues of the older adult population have increasingly garnered widespread attention. The acceleration of this process has already posed a significant impact on the planning and provision of health and social care systems on a global scale (1). China possesses the world’s largest older adult population; research projects that by 2050, the population aged 65 and above in China will reach 395 million (2). This trend of a continuously increasing older population underscores the necessity of a deep understanding of the internal and external change processes in older adults, which is of critical significance for promoting their active social integration and preventing the transition into frailty or disease states.

Frailty is a widely recognized clinical syndrome, primarily characterized by increased vulnerability and a reduced capacity to cope with stressors resulting from decreased physiological reserves or multisystem dysregulation in older adults, involving physiological, psychological, and social dimensions (3). Among the Chinese older adult population, the prevalence of frailty is notably prominent and gradually increases with advancing age (4). Frailty is a significant risk factor for various adverse health outcomes in older adults, including an increased risk of falls and disability (5). In China’s community settings, approximately 10% of older adults aged 60 and above, 15% of those aged 75–84, and nearly 25% of those aged 85 and above are affected by frailty (6), indicating that the prevalence of frailty among the community-dwelling older population is at a relatively high level. It not only compromises the health status, functional independence, and quality of life of older adults but also imposes significant caregiving pressure and economic burdens on families and society (7). These features make it important to examine modifiable psychological and cognitive correlates within longitudinal designs. Examine modifiable psychological and cognitive correlates within longitudinal designs.

Frailty constitutes a constellation of symptoms involving complex interactions among diverse factors. Recent studies by Soysal et al. (8, 9) indicate that depression and frailty share underlying pathological mechanisms, including inflammation (e.g., IL-6, CRP), oxidative stress, and mitochondrial dysfunction. Depression precipitates sedentary behavior, diminished appetite, and physiological depletion, thereby serving as a potent accelerator of physical frailty; these two conditions often form a “bidirectional vicious cycle.” Frailty is not only a physical syndrome but is also shaped by demographic, clinical, psychological, and social factors. Previous studies have shown that age, sex, education, multimorbidity, cognitive impairment, depressive symptoms, physical activity, and social determinants are associated with frailty risk or progression (4, 9–12). Accordingly, this study included sociodemographic characteristics, chronic disease burden, and cognitive function as covariates in the longitudinal analyses. Research has also revealed that cognitive status and frailty mutually influence one another within the cycle of aging-related functional decline (13), and that frailty is associated with poorer cognitive performance (14). Furthermore, according to the dynamic equilibrium hypothesis of frailty, frailty is a potentially reversible process wherein the status may either improve or deteriorate over time (15). Therefore, given the high prevalence and ubiquity of frailty, along with its associated adverse health consequences and influencing factors, it is imperative to explore modifiable factors and underlying mechanisms related to the progression of frailty, with the aim of reversing the trend of frailty in older adults.

Self-perceptions of aging (SPA) refer to the subjective perceptions and emotional responses elicited in older adults when confronted with physiological, psychological, and social threats associated with aging. Grounded in Stereotype Embodiment Theory (SET), age-related stereotypes–whether positive or negative–that individuals internalize as they age eventually become self-relevant and transform into personal expectations and views regarding their own aging process (16). These internalized beliefs constitute self-perceptions of aging and appear to exert influence through multiple psychological, behavioral, and physiological pathways (16) Mood Congruency Theory further suggests that current affective states shape the selection and interpretation of self-referential information; thus, depressive mood may make aging-related losses more cognitively salient. Existing research indicates that more negative self-perceptions of aging are associated with a range of adverse health outcomes, including frailty, falls, hospitalization, and a decline in activities of daily living (17); they are also correlated with a higher risk of mortality, shortened lifespan (18), more pronounced depressive symptoms (19), and poorer cognitive function (20). Joshanloo (21) challenged the traditional unidirectional perspective through rigorous longitudinal analysis, finding that the predictive effect of depressive symptoms on self-perceptions of aging was even stronger than the prediction of depression by perceptions of aging. This study supports the “mood-congruency effect,” wherein older adults experiencing depression tend to selectively attend to and process negative information, thereby forming distorted and catastrophic assessments of their own aging status. Furthermore, Fernandes-Pires et al. (22), utilizing a dyadic coping framework, found that negative aging perceptions weaken coping resources between older couples, thereby exacerbating depressive symptoms and revealing the socio-interpersonal nature of this interaction. For older adults with compromised health status, attitudes toward aging may become more negative, potentially further increasing their health risks (23).

Based on a sample of community-dwelling older adults, this study conducts an in-depth analysis of the relationship between self-perceptions of aging and frailty, thereby enhancing the understanding of the internal mechanisms that may accelerate or delay the frailty process. Existing research indicates that frailty is closely associated with multiple psychological, cognitive, and physical indicators, such as depressive symptoms (9) and cognitive status (24). Depression is one of the most common mental health concerns in old age and is regarded as a prodromal manifestation of frailty (25). Maintaining a high level of mental health may help prevent frailty or delay its onset, which is of critical importance (26). Therefore, given that depressive symptoms are closely associated with both self-perceptions of aging and frailty, they may also serve as potential psychological and cognitive pathways through which self-perceptions of aging influence frailty.

In summary, grounded in Psychosomatic Interaction Theory (PIT) and SET, this study conducts a longitudinal survey of older adults and constructs a cross-lagged panel model to clarify the relationships and underlying mechanisms linking self-perceptions of aging, depression, and frailty. PIT is consistent with the biopsychosocial model, which emphasizes that biological, psychological, and social processes interact in health and disease (27); it therefore offers a framework for understanding how frailty may trigger depressive symptoms and how depression may in turn accelerate physical decline. SET explains how internalized aging stereotypes become self-relevant and influence aging-related cognition, behavior, and health. Although previous studies have explored the relationships among frailty, depression, and self-perceptions of aging, the majority have relied on cross-sectional designs, limiting the ability to infer directional pathways. To address this methodological gap, our study utilized a three-wave cross-lagged panel model (CLPM) to examine the dynamic, longitudinal interactions among these variables. Furthermore, by focusing on community-dwelling older adults in China, this study provides evidence for community-based public health interventions and nursing practices, where early screening and continuous follow-up are more feasible than in institutional or acute-care settings. We aim to test the following three hypotheses:

*H1*: Frailty, depression, and self-perceptions of aging in community-dwelling older adults are significantly positively correlated at different time points, and each variable demonstrates significant cross-temporal stability.

*H2*: Significant bidirectional cross-lagged associations exist between frailty and depression, and between depression and self-perceptions of aging; specifically, frailty at a preceding time point predicts depression at a subsequent time point, and vice versa; depression at a preceding time point predicts negative aging perceptions at a subsequent time point, and vice versa.

*H3*: Depression mediates the longitudinal association between frailty and self-perceptions of aging. That is, frailty is expected to influence negative self-perceptions of aging indirectly through depressive symptoms; depression serves as a core hub connecting physical function with self-perceptions of aging.

This study further aimed to elucidate the unique mechanism of depression, hypothesizing it as a critical psychosomatic hub that may mediate the longitudinal association between physical frailty and self-perceptions of aging.

## Materials and methods

### Participants and samples

Older adults who voluntarily participated in physical examinations in communities such as Xinglong and Binhu in Xinxiang City, Henan Province, from March 2023 to December 2025 were recruited as study participants. Three waves of data collection were conducted at approximately one-year intervals. The inclusion criteria were: (1) aged 65 years or older; and (2) provided signed informed consent and voluntary participation in the study. The exclusion criteria were: (1) a diagnosis of dementia; and (2) inability to complete data collection due to severe mental disorders, language or hearing impairments, or other reasons. This study was reviewed and approved by the Ethics Committee of Henan Medical University (Approval Number: XYLL-20240030), and all participants provided written informed consent before participation.

### Research tools

#### Socio-demographic variables

This researcher-developed form was designed based on a systematic review of relevant literature and consultations with experts in geriatric nursing. Its items encompassed demographic characteristics (age, gender, height, weight, educational level, living arrangements, personal monthly income, and marital status) and health behaviors and conditions (smoking, drinking, and types of chronic diseases). Based on prior evidence concerning frailty-related demographic, clinical, psychological, cognitive, and social factors (4, 9–14), sex, age, education level, number of chronic diseases, and cognitive function were treated as covariates in subsequent analyses.

#### Self-perceptions assessment

The B-APQ used in this study is the brief version originally developed by Sexton et al. (28) and culturally adapted into Chinese by Wang et al. (29). The scale comprises five dimensions: timeline-chronic, positive consequences, positive control, negative consequences and control, and emotional representation. Items are assessed using a 5-point Likert scale. The total score is calculated by summing the scores of all items, ranging from 17 to 85, with higher scores indicating more negative attitudes toward one’s own aging process.

#### Depressive symptoms assessment

The Patient Health Questionnaire-9 (PHQ-9) (30) is currently one of the preferred instruments for screening depression. The scale consists of 9 items, each rated on a 4-point scale: “not at all,” “several days,” “more than half the days,” and “nearly every day,” scored as 0, 1, 2, and 3, respectively. A score of ≤4 indicates no depression; 5–9 indicates possible mild depression; 10–14 indicates possible moderate depression; 15–19 indicates possible moderately severe depression; and ≥20 indicates possible severe depression.

#### Frailty assessment

Assessment was conducted using the Fried Frailty Phenotype scale (31). This scale encompasses five dimensions: unintentional weight loss, self-reported exhaustion, weakness (decreased grip strength), slowness (slow walking speed), and low physical activity level. Unintentional weight loss was defined as losing 4.5 kg or more than 5% of baseline body weight unintentionally within the past year, assessed via self-report. Exhaustion was assessed using two items from the Center for Epidemiologic Studies Depression Scale (CES-D): “I felt that everything I did was an effort” and “I could not get going” in the past week. Grip strength was measured using an electronic hand dynamometer, with participants tested three times and the maximum value recorded; weakness was determined using gender- and body mass index (BMI)-specific thresholds: for males, ≤29 kg for BMI ≤ 24, ≤30 kg for BMI 24.1–26, ≤30 kg for BMI 26.1–28, and ≤32 kg for BMI > 28; for females, ≤17 kg for BMI ≤ 23, ≤17.3 kg for BMI 23.1–26, ≤18 kg for BMI 26.1–29, and ≤21 kg for BMI > 29. Slowness was evaluated by measuring the time required for participants to walk 4.57 meters. Walking speed was classified as slow based on established height and gender standards: for males, height ≤173 cm with time ≥7 s, or height >173 cm with time ≥6 s; for females, height ≤159 cm with time ≥7 s, or height >159 cm with time ≥6 s. Low physical activity level was assessed using the International Physical Activity Questionnaire-Short Form (IPAQ-SF) (32). Each dimension was scored as 1 point, yielding a total score range of 0–5, with higher scores indicating greater severity of frailty. Based on the total score, participants’ frailty status was classified as robust (0 points), pre-frail (1–2 points), and frail (3–5 points).

#### Covariates

Sociodemographic characteristics collected at baseline included sex, age, education level (junior high school or below / high school or above), and the number of self-reported chronic conditions (0–1 type / 2 types or more). Cognitive function was measured using the Montreal Cognitive Assessment (MoCA) (33). “The total score ranges from 0 to 30, and scores lower than 26 suggest cognitive impairment of different severities.” Because complete MoCA data were available at T2 rather than baseline, the T2 MoCA score was included as a proxy covariate for cognitive function together with educational attainment. This temporal mismatch was considered when interpreting the adjusted longitudinal estimates. Questionnaire-based variables were collected through self-administered questionnaires, whereas grip strength and walking speed were assessed through standardized physical measurements.

### Statistical analysis

Descriptive statistical analyses were performed using SPSS version 26.0. Continuous variables conforming to a normal distribution (e.g., age, frailty, depression, self-perceptions of aging, and cognitive function scores) were expressed as mean ± standard deviation (Mean ± SD); categorical variables (e.g., gender, education level) were described using frequencies and percentages (*n*, %). Pearson correlation analysis was employed to examine pairwise associations among the three core variables across waves, while partial correlation analysis controlling for sex, age, education level, number of chronic diseases, and cognitive function was used to examine associations between baseline covariates and the core variables, providing a basis for subsequent model construction.

Methodological research indicates that the Cross-Lagged Panel Model (CLPM) effectively elucidates temporal effects among variables in longitudinal data and examines the temporal direction of predictive associations (34). In this study, Mplus version 8.3 was used to construct the CLPM to investigate the mutual predictive relationships among frailty, depression, and self-perceptions of aging over time. The specific steps for model construction were as follows: frailty, depression, and self-perceptions of aging at three time points (T1, T2, T3) were included in the model, allowing for correlations between residuals of variables at the same time point, and specifying autoregressive paths and cross-lagged paths between adjacent time points. Simultaneously, to reduce potential confounding factors, age, gender, education level, the number of chronic diseases, and cognitive function were included in the analysis as covariates to control for their influence on the core variables at each time point.

Model fit was comprehensively evaluated using the Chi-square value (χ2), degrees of freedom (df), and multiple fit indices, including the Comparative Fit Index (CFI), Tucker-Lewis Index (TLI), Root Mean Square Error of Approximation (RMSEA), and Standardized Root Mean Square Residual (SRMR). According to conventional standards for structural equation modeling, model fit is considered good or acceptable when CFI > 0.90 (or acceptable > 0.80), TLI > 0.90 (or acceptable > 0.80), RMSEA < 0.08, and SRMR < 0.08 (35, 36). Data integrity was verified prior to analysis, and there were no missing values for demographic, clinical, or symptom variables in the final dataset. The data underlying this article will be shared on reasonable request to the corresponding author.

## Results

### Descriptive statistics of study variables

As presented in Table 1, across the three measurement waves, the mean scores for frailty were 1.15, 0.79, and 0.84, respectively; the mean scores for depression were 2.22, 1.19, and 2.14, respectively. Self-perceptions of aging were assessed based on total scores, with mean values of 39.77, 40.82, and 37.90, respectively.

**Table 1:** Descriptive statistics of core variables.

Variables	Time	M	SD	Skewness	Kurtosis
Fried	T1	1.15	1.01	0.82	0.50
	T2	0.79	0.78	0.69	−0.16
	T3	0.84	0.87	0.82	0.09
Dep	T1	2.22	2.95	1.90	5.14
	T2	1.19	1.93	2.47	7.91
	T3	2.14	2.62	2.70	16.01
SPA	T1	39.77	8.71	0.48	1.00
	T2	40.82	6.44	0.42	0.25
	T3	37.90	10.70	0.14	−0.37

The results of normality testing indicated that the absolute values of skewness coefficients for all variables were less than 3. Although the kurtosis coefficient for depression at T3 was slightly elevated, given that Maximum Likelihood (ML) estimation possesses a certain degree of robustness against deviations from normality, and considering the large sample size, the data distribution fundamentally satisfied the analytical requirements for structural equation modeling.

### Correlation analysis of core variables

Partial correlation analysis controlling for covariates showed that age was significantly positively correlated with self-perceptions of aging (*r* = 0.44); cognitive function was significantly negatively correlated with frailty (*r* = −0.21), depression (*r* = −0.18), and self-perceptions of aging (*r* = −0.28), indicating that better cognitive function is associated with a more favorable psychosomatic profile. Among the core variables, baseline frailty was significantly positively correlated with both baseline depression (*r* = 0.32) and self-perceptions of aging (*r* = 0.31), supporting subsequent longitudinal modeling (see Table 2).

Pearson correlation analysis was employed to examine the pairwise correlations among frailty, depression, and self-perceptions of aging across the three time points. As shown in Table 3.

**Table 3:** Pearson correlation matrix among variables (fried, frailty; dep, depression; SPA, self-perceptions of aging; T1–T3, waves 1–3).

Variables	1	2	3	4	5	6	7	8	9
1. Fried T1	1.00								
2. Fried T2	0.29**	1.00							
3. Fried T3	0.30**	0.41**	1.00						
4. Dep T1	0.32**	0.03	0.14*	1.00					
5. Dep T2	0.22**	0.09	0.13*	0.24**	1.00				
6. Dep T3	0.20**	0.07	0.13*	0.31**	0.47**	1.00			
7. SPA T1	0.31**	0.16**	0.25**	0.33**	0.06	0.12*	1.00		
8. SPA T2	0.22**	0.19**	0.20**	0.35**	0.29**	0.25**	0.25**	1.00	
9. SPA T3	0.22**	0.13*	0.18**	0.28**	0.16**	0.33**	0.19**	0.31**	1.00

*p < 0.05, **p < 0.01, ***p < 0.001.

*Cross-temporal stability*: The same variable exhibited significant positive correlations between adjacent time points. For instance, frailty at T1 was significantly positively correlated with frailty at T2 (*r* = 0.29, *p* < 0.01), and depression at T1 was significantly positively correlated with depression at T2 (*r* = 0.24, *p* < 0.01), indicating that each variable possesses relative stability over time.

*Inter-variable correlations*: Frailty, depression, and self-perceptions of aging were mostly significantly positively correlated with each other at each time point. Specifically, frailty at T1 was significantly correlated with concurrent depression (*r* = 0.32, *p* < 0.01) and self-perceptions of aging (*r* = 0.31, *p* < 0.01); depression at T1 also demonstrated significant longitudinal correlations with self-perceptions of aging at T2 (*r* = 0.35, *p* < 0.01) and T3 (*r* = 0.28, *p* < 0.01).

The results of the correlation analysis indicate close statistical associations among the variables, supporting subsequent examination using a cross-lagged panel model.

**Table 2:** Correlation analysis between covariates and core variables (MoCA, montreal cognitive assessment).

Variable	Age	Sex	Education	Chronic diseases	Cognition a
T1 Frailty	0.17**	0.08	−0.07	0.06	−0.21**
T2 Frailty	0.27**	0.07	−0.06	0.13*	−0.20**
T3 Frailty	0.32**	0.04	−0.13*	0.08	−0.06
T1 Dep	−0.02	−0.08	−0.13*	0.21**	−0.18**
T2 Dep	−0.10	−0.18**	0.02	0.18**	0.06
T3 Dep	−0.10	−0.13*	0.05	0.25**	0.07
T1 SPA	0.44**	0.08	−0.10	0.18**	−0.28**
T2 SPA	0.05	0.02	−0.11	0.15*	−0.14*
T3 SPA	0.07	0.11	0.02	0.14*	−0.10

*p < 0.05, **p < 0.01, ***p < 0.001.

### Cross-lagged panel analysis of frailty, depression, and self-perceptions of aging

To examine the temporal predictive relationships among the variables, a cross-lagged panel model was constructed in this study. The model incorporated frailty, depression, and self-perceptions of aging at three time points (T1, T2, T3), allowing for correlations between residuals of variables at the same time point, while controlling for the effects of age, gender, education level, number of chronic diseases, and cognitive function.

### Evaluation of model fit

The fit indices for the CLPM constructed in this study are presented in Table 4. The Chi-square value (*χ^2^*) was 49.583, with 27 degrees of freedom (*df*). The Chi-square to degrees of freedom ratio (*χ^2^/df*) was 1.84, which is well below the recommended threshold of 3, indicating excellent model parsimony. The Root Mean Square Error of Approximation (RMSEA) was 0.054, with a 90% confidence interval (95%*CI*) of [0.029, 0.077], indicating acceptable close fit. The Comparative Fit Index (CFI) was 0.960, exceeding 0.90. The Standardized Root Mean Square Residual (SRMR) was 0.037, well below the critical threshold of 0.08.

**Table 4:** Fit indices of the cross-lagged panel model.

Fit indices	Statistic	Reference criteria
*χ*^2^ (Chi-Square)	49.583	—
df (Degrees of Freedom)	27	—
*χ*^2^/df	1.84	<3
CFI	0.960	>0.90
TLI	0.855	>0.90
RMSEA	0.054	<0.05
RMSEA 90% CI	[0.029, 0.077]	—
SRMR	0.037	<0.08

Notably, the Tucker-Lewis Index (TLI) was 0.855, slightly below the conventional standard of 0.90. Regarding this, Kenny noted that the TLI imposes a heavy penalty for model complexity and correlations, and it is prone to underestimation, particularly in cases involving moderate sample sizes and models containing multiple time points and covariates. Considering the acceptable RMSEA and SRMR values and the *χ2/df* ratio, the overall model fit was considered acceptable; however, the below-threshold TLI should be considered when interpreting the results.

### Path coefficient analysis

**Figure 1:**
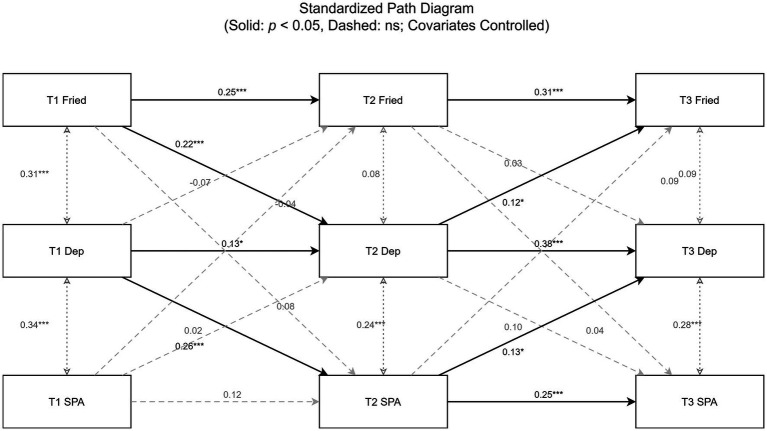
Path diagram of the cross-lagged panel model of frailty, depression, and self-perceptions of aging. Solid arrows indicate statistically significant paths (*p* < 0.05), whereas dashed arrows represent non-significant paths. Curved arrows denote concurrent covariances between variables at each time point. Standardized path coefficients are reported along the paths. Fried, frailty; Dep, depression; SPA, self-perceptions of aging; T1–T3, waves 1–3.*p < 0.05, **p < 0.01, ***p < 0.001.

The path coefficients of the model and the results of significance testing are presented in Table 5 and Figure 1.

1. Autoregressive paths (Stability).

**Table 5:** Analysis of cross-lagged path coefficients (Fried, frailty; Dep, depression; SPA, self-perceptions of aging; Est., estimate; S.E., standard error; C.R., critical ratio; T1-T3, waves 1–3).

Path	Est.	S.E.	C.R. (t)	*p*	*β*
Autoregressive paths					
T1·Fried → T2·Fried	0.195	0.046	4.243	***	0.252
T2·Fried → T3·Fried	0.343	0.061	5.623	***	0.308
T1 Dep → T2 Dep	0.086	0.041	2.110	*	0.131
T2 Dep → T3·Dep	0.516	0.075	6.904	***	0.381
T1·SPA → T2·SPA	0.086	0.049	1.738	0.082	0.116
T2·SPA → T3·SPA	0.416	0.099	4.204	***	0.250
Cross-lagged Paths					
(T1 → T2, T2 → T3)					
T1 → T2					
T1·Fried → T2·Dep	0.419	0.113	3.689	***	0.219
T1·Fried → T2·SPA	0.520	0.378	1.374	0.169	0.082
T1·Dep → T2·Fried	−0.018	0.016	−1.095	0.273	−0.068
T1·Dep → T2·SPA	0.567	0.136	4.179	***	0.260
T1·SPA → T2·Fried	−0.003	0.006	−0.555	0.579	−0.037
T1·SPA → T2·Dep	0.005	0.015	0.359	0.720	0.024
T2 → T3					
T2·Fried → T3·Dep	0.092	0.181	0.507	0.612	0.027
T2·Fried → T3·SPA	0.559	0.809	0.691	0.489	0.041
T2·Dep → T3·Fried	0.053	0.025	2.096	*	0.118
T2·Dep → T3·SPA	0.530	0.335	1.582	0.114	0.096
T2·SPA → T3·Fried	0.012	0.007	1.561	0.118	0.086

*p < 0.05, **p < 0.01, ***p < 0.001.

With the exception of the T1 to T2 path for self-perceptions of aging (SPA), all variables exhibited a certain degree of cross-temporal stability. Specifically, T1 frailty significantly predicted T2 frailty (*β* = 0.252, *p* < 0.001), and T2 frailty significantly predicted T3 frailty (*β* = 0.308, *p* < 0.001); T1 depression significantly predicted T2 depression (*β* = 0.131, *p* = 0.034), and T2 depression significantly predicted T3 depression (*β* = 0.381, *p* < 0.001). T2 self-perceptions of aging significantly predicted T3 self-perceptions of aging (*β* = 0.25, *p* < 0.001); however, the autoregressive path from T1 to T2 did not reach the level of significance (*p* = 0.081). This suggests that aging cognition during this phase may be more subject to the dynamic impact of external variables, such as depressive mood, rather than being maintained solely by inertia.

1. Cross-lagged paths (temporal predictive associations).

After controlling for autoregressive effects and covariates, the cross-lagged relationships among the variables exhibited interaction characteristics centered on depression:

The results of this study revealed significant bidirectional temporal associations among the variables. At the level of “psychosomatic interaction,” frailty and depression showed a reciprocal predictive pattern: baseline frailty significantly and positively predicted midterm depression (*β* = 0.219, *p* < 0.001), indicating that greater physical frailty preceded higher subsequent psychological distress; conversely, midterm depression significantly and positively predicted subsequent frailty (*β* = 0.118, *p* = 0.035), indicating that depressive symptoms preceded subsequent increases in frailty in later stages. Simultaneously, at the level of “cognition-emotion interaction,” the two variables exhibited a spiraling pattern of “emotion driving cognition — cognition reacting upon emotion.” Baseline depression demonstrated strong predictive power for midterm self-perceptions of aging (*β* = 0.26, *p* < 0.001), indicating that negative emotion may contribute to subsequent negative views on aging. Furthermore, the negative aging conceptions formed in the midterm acted as a cognitive stressor, significantly predicting subsequent depressive symptoms (*β* = 0.125, *p* = 0.021). At the level of “frailty and self-perceptions of aging interaction,” the direct cross-lagged paths between frailty and self-perceptions of aging in the model were not significant (*p* > 0.05). These findings are consistent with the hypothesis that depression may constitute an indirect pathway between frailty and self-perceptions of aging; however, a formal indirect-effect test is required to establish mediation.

### Variance explained (*R*^2^)

The model demonstrated good explanatory power for the variance of each endogenous variable. After incorporating variables from the preceding time point and covariates, the model accounted for: 27.6% of the variance in T3 depression (R^2^ = 0.276, *p* < 0.001); 25.1% of the variance in T3 frailty (R^2^ = 0.251, *p* < 0.001); and 13.3% of the variance in T3 self-perceptions of aging (R^2^ = 0.133, *p* < 0.001). This indicates that the psychosomatic interaction model constructed in this study effectively explains the temporal changes in the health status of older adults (see Table 6).

**Table 6:** Variance explained for endogenous variables (fried, frailty; dep, depression; SPA, self-perceptions of aging; T1–T3, waves 1–3).

Variables	Time	R^2^	*p*
Frailty (Fried)	T2	0.165	<0.001
	T3	0.251	<0.001
Depression (Dep)	T2	0.165	<0.001
	T3	0.276	<0.001
Self-perceptions of aging (SPA)	T2	0.165	<0.001
	T3	0.133	<0.001

## Discussion

### The driving role of depression on self-perceptions of aging

The cross-lagged analysis in this study revealed a critical finding: baseline depression significantly and positively predicted negative self-perceptions of aging at T2 (*β* = 0.260, *p* < 0.001). This result suggests that depression is not merely a “consequence” of psychosomatic decline in older adults, but rather a “core driver” promoting the development of negative aging experiences. This aligns with the recent longitudinal analysis of data from the German Ageing Survey by Joshanloo (21), which similarly confirmed that depressive symptoms serve as prospective predictors of negative views on aging (e.g., an increased focus on physical deficits) among older adults, and that this influence accumulates within individuals over time.

The phenomenon of “aging driven by sadness” can primarily be explained mechanistically through Mood Congruency Theory. As a negative emotional state, depression activates negative schemas in the brains of older adults, leading to selective biases in the processing of self-referential information (37). Recent neuropsychological research indicates (38) that depressive symptoms significantly attenuate the “positivity effect” in older adults, predisposing them to focus more on negative information during memory retrieval and cognitive processing. Older adults in a depressive state often scrutinize themselves through a “gray filter,” remaining hypersensitive to minor physiological declines in daily life—such as occasional forgetfulness or decreased physical stamina—and catastrophically interpreting them as irreversible signals of aging rather than temporary health fluctuations. This cognitive bias predisposes them to retrieve memory cues consistent with the “aging as loss” stereotype when evaluating their own aging process, thereby reinforcing negative self-perceptions of aging.

Furthermore, from the defense perspective of Stereotype Embodiment Theory, depressive mood may undermine the psychological defenses of older adults against social stereotypes (39). Although SET emphasizes that the internalization of stereotypes leads to declines in physical and mental health, recent studies have begun to focus on the reverse pathway of this process. Research by Fernandes-Pires et al. (22) indicates that a scarcity of psychological resources, such as high levels of depression, reduces self-efficacy in older adults, rendering them more susceptible to the erosion caused by ubiquitous “ageism” and negative aging concepts in society. In other words, depression depletes the psychological resilience resources of older adults and accelerates the internalization process from “social stereotypes” to “self-perceptions of aging,” thereby making it easier for older adults to “adopt” those negative aging labels.

This study confirms the pivotal hub role of depression within the psychosomatic interaction perspective: it reversely reshapes older adults’ self-perceptions of aging through the dual pathways of cognitive bias (mood congruency) and defense weakening (reverse perspective of SET). These findings suggest to community nursing practitioners that during mental health management for older adults, alleviating depressive mood not only enhances the immediate emotional state but also serves as an effective target for blocking the formation of negative aging cognitions, holding significant importance for breaking the vicious cycle of “depression–aging perception” (40).

### The dynamic interaction between frailty and depression

This study confirms a significant bidirectional predictive relationship between frailty and depression through the cross-lagged panel model, revealing a close dynamic joint mechanism between the “body” and “mind” of older adults. Specifically, baseline frailty significantly predicted midterm depressive mood (*β* = 0.219, *p* < 0.001), while midterm depressive mood predicted the aggravation of subsequent frailty (*β* = 0.118, *p* < 0.05). This finding is highly consistent with the results of a recent longitudinal study by Soysal et al. (9) focusing on community-dwelling older adults aged over 70, which similarly found that frailty and depression are temporally associated in both directions, and their co-occurrence significantly accelerates the deterioration of overall health in older adults.

This bidirectional association primarily reflects the “somatopsychic” effect within Psychosomatic Interaction Theory. As the severity of frailty increases, older adults face physiological limitations such as slowed gait, decreased grip strength, and physical exhaustion; the loss of these somatic functions directly impairs their ability to maintain independence in daily living. Research by Alhwoaimel et al. (41) indicates that older adults with comorbid frailty are more inclined toward sedentary behavior, and their social participation and quality of life are significantly lower than those of their healthy peers. When older adults perceive that they can no longer control their bodies or participate in social interactions as they used to, this “loss of sense of control” easily translates into psychological feelings of frustration and helplessness, thereby inducing or exacerbating depressive mood. Therefore, frailty is not only a “collapse” of physiological function but also a “stressor” for mental health.

Conversely, the predictive effect of depression on subsequent frailty reveals the pathogenic mechanism of “psychological illness affecting the physical body.” As a negative emotional state, depression “erodes” physical health through dual behavioral and physiological pathways. At the behavioral level, depression is often accompanied by a lack of motivation and reduced physical activity, causing older adults to fall into a vicious cycle of “sedentariness–muscle loss,” accelerating the process of physical frailty (41). Regarding deeper physiological mechanisms, the study by Jiang et al. (42) provides a biological explanation for these results: they found that depressive symptoms are closely associated with elevated levels of systemic inflammation, such as C-reactive protein and neutrophils, as well as reductions in volume in specific brain regions, such as gray matter; these inflammatory factors act as mediating variables, directly participating in the pathological process from depression to physical frailty. It can thus be inferred that chronic depressive mood causes substantial “wear and tear” on physical function by activating the stress response of the neuro-immune system.

Based on these findings, frailty and depression appear to be closely interrelated clinical syndromes rather than independent conditions. Physical weakness may restrict daily functioning and social participation, while depressive symptoms may further accelerate behavioral and physiological pathways related to frailty. This finding suggests that clinical practice should adopt a strategy of integrated physical and psychological care: for frail older adults, it is necessary not only to conduct physical rehabilitation but also to monitor relevant health indicators and implement psychological interventions to reduce the risk of a vicious cycle of psychosomatic interaction.

### The mediating hub effect of depression and pathway blockade

A key finding from the cross-lagged analysis in this study is that there is no direct longitudinal predictive path between frailty and self-perceptions of aging; while the observed pattern is consistent with an indirect pathway through depression, namely frailty to depression to self-perceptions of aging. This result highlights the potentially important role of depression within the psychosomatic health network of older adults, indicating that objective decline in physical function may not directly translate into negative subjective perceptions of aging; rather, emotional status plays a critical transformative and regulatory role therein. This aligns with the results of structural equation modeling analysis of multidimensional health data for community-dwelling older adults by Geng et al. (43), which noted that psychological distress is an essential pathway connecting somatic impairment and subjective health evaluation.

Based on the holistic view of Psychosomatic Interaction Theory, this hypothesized indirect pathway illustrates the specific pathway through which physical symptoms transform into psychological cognition. From the perspective of somatic illness affecting the mind, frailty, as a physiological stressor, first disrupts older adults’ sense of physical balance and control, triggering anxiety and depressive moods; subsequently, this negative emotion becomes the “internal catalyst” described in Stereotype Embodiment Theory (SET) (16), making older adults more prone to attributing physical weakness to “irreversible aging” rather than merely “illness” or “fatigue” (44). In other words, depressive mood acts as a “negative amplifier,” magnifying physiological frailty signals into psychological aging panic. Recent research also confirms (45) that after controlling for depressive symptoms, the influence of objective physical functional impairment on subjective aging experience in older adults decreases by nearly 40%, providing additional support for the potential emotional pathway.

It is worth noting that the direct path from frailty to self-perceptions of aging was not significant in the present longitudinal model. Rather than indicating a complete disconnection between SPA and frailty, this finding suggests that their association may be conditional, indirect, or more evident in cross-sectional designs than in adjusted longitudinal pathways. Previous studies have reported associations between negative SPA and frailty, but many of these studies used cross-sectional data or did not fully account for depressive symptoms. In our community-dwelling sample, older adults may have retained compensatory resources, social participation, and access to community support, which could buffer the immediate cognitive impact of physical frailty. Thus, the non-significant direct path should be interpreted as evidence that depressive symptoms are a key intervening mechanism in this dataset, not as evidence that SPA and frailty are unrelated in all contexts. However, if depressive mood is allowed to develop unchecked, it will not only deepen the sense of aging but also further exacerbate physical frailty by activating neuroimmune mechanisms such as immune pathways, forming a closed loop (46). Research by Vaughan et al. (47) reveals that systemic inflammation induced by depression, such as elevated IL-6 and CRP, serves as an important biological mediator accelerating the onset of sarcopenia in frail older adults, thereby biologically confirming the self-fulfilling prophecy of aging in older adults.

In summary, depression not only connects “body” and “mind” but also serves as the key “valve” to block vicious psychosomatic interactions. For community nursing personnel, this finding suggests a shift in the focus of intervention strategies: when managing frailty issues in older adults, physical interventions alone may be insufficient; it is imperative to synchronously identify and intervene in depressive mood through psychological screening (e.g., the GDS scale). Based on the aforementioned “frailty–depression–frailty” vicious cycle mechanism, future nursing interventions should prioritize breaking this psychosomatic interaction chain. Cognitive Behavioral Therapy (CBT) or Mindfulness-Based Stress Reduction (MBSR) targeting depression may represent potentially useful approaches for addressing this pathway. Specifically, CBT can help identify and correct the catastrophic thinking of “equating physical weakness with irreversible aging” through cognitive restructuring techniques (48), thereby blocking the internalization process of negative aging stereotypes. Meanwhile, mindfulness-based interventions may complement cognitive approaches by promoting non-judgmental acceptance of current bodily sensations and reducing psychological distress. Further intervention studies are needed to determine whether these approaches alter subsequent frailty or inflammatory processes.

From a public health and clinical nursing perspective, our findings highlight the urgent need for integrated care models in community settings. Community-dwelling older adults often differ from those in hospitals or long-term care institutions because they may have greater independence, more varied social participation, and earlier-stage health decline; these contextual features may help explain why frailty did not directly predict SPA after depression and covariates were considered. In non-community settings, where illness severity, functional dependence, or institutional stressors may be greater, the relationship among frailty, depression, and SPA may show different patterns and warrants further comparison. Given that the observed findings are consistent with a potentially important indirect role of depression in the association between physical frailty and self-perceptions of aging, community nurses and primary care providers should prioritize regular psychological screening alongside routine physical assessments. Implementing early, targeted interventions--such as Cognitive Behavioral Therapy (CBT), mindfulness-based stress reduction, or community peer-support groups--may help reduce the vicious cycle of physical decline and negative aging cognition. Furthermore, health education programs should be culturally tailored to empower older adults, helping them reframe their aging experiences and maintain psychological resilience despite physical vulnerabilities. The use of T2 rather than baseline MoCA as a proxy covariate should also be considered when interpreting the adjusted longitudinal estimates.

## Conclusion

This longitudinal study elucidates the dynamic psychosomatic interactions among frailty, depression, and self-perceived aging in community-dwelling older adults. Our findings support a bidirectional temporal association between physical frailty and depressive symptoms, as well as reciprocal temporal associations between depression and negative self-perceived aging. Crucially, this study identifies depression as a potentially important intervening factor in the pathway from physiological frailty to subjective aging cognition; the observed pattern is consistent with an indirect pathway from physical frailty to negative self-perceptions of aging through depressive symptoms. These findings underscore the critical importance of mental health in the management of frailty. For community nursing practice, interventions should shift from a purely physical focus to a holistic psychosomatic approach. Routine screening and timely interventions for depressive symptoms--even at subthreshold levels--are essential. By targeting depression, healthcare providers may help reduce the maladaptive psychosomatic loop, thereby delaying physical decline and fostering a more positive aging experience.

## Data Availability

The raw data supporting the conclusions of this article will be made available by the authors, without undue reservation.
